# Evidence for deprescription in primary care through an umbrella review

**DOI:** 10.1186/s12875-020-01166-1

**Published:** 2020-06-08

**Authors:** Antonio Olry de Labry Lima, Jorge Marcos Marcos, Alfonso Marquina Marquez, María de los Ángeles González Vera, Antonio Matas Hoces, Clara Bermúdez Tamayo

**Affiliations:** 1 Andalusian School of Public Health (Spanish acronym EASP), Granada, Spain; 2 Instituto de Investigación Biosanitaria ibs, GRANADA, Granada, Spain; 3 CIBER en Epidemiología and Salud Pública (CIBERESP), Madrid, Spain; 4 Public Health Research Group, University of Alicante, Alicante, Spain; 5 Faculty of Education and Social Work, University of Valladolid, Valladolid, Spain; 6 GIESA, Applied Sociocultural Studies (SEJ208), University of Granada, Granada, Spain; 7 Farmacia del Puente (Pinos Puente), Granada, Spain; 8 Centro Andaluz de Información del Medicamento (CADIME), Campus Universitario de Cartuja, Granada, Spain

**Keywords:** Primary care, Deprescription, Intervention, Umbrella review

## Abstract

**Background:**

There is a high prevalence of potentially inappropriate prescriptions in primary care. This is associated with more frequent adverse events, lower quality of life and more frequent visits to hospital accident & emergency departments. The aim of the present study is to summarise available evidence on the effectiveness of deprescription interventions in primary care, and to describe the barriers and enablers of the process from the point of view of patients and healthcare professionals.

**Methods:**

We designed an umbrella review which includes nine systematic reviews. More than 50% of included studies were performed with adults in primary care. Two reviewers independently performed data extraction and analysis.

**Results:**

In considering studies of the effectiveness of interventions, it can be observed that the educational component of deprescription procedures is a key factor, whilst procedures tailored towards the patient’s situation offer better results. With regards to studies involving healthcare professionals, the main explored areas were the balance between risks and benefits, and the need to improve communication with patients as well as other colleagues involved in patient care. Amongst the identified barriers we found lack of time, inability to access all information, being stuck in a routine, resistance to change and a lack of willingness to question the prescription decisions made by healthcare colleagues. With regards to patients, it is clear that they have worries and doubts. In order to overcome these issues, a good relationship with healthcare professionals and receipt of their support is required during the process.

**Conclusions:**

Optimizing medication through targeted deprescribing is an important part of managing chronic conditions, avoiding adverse effects and improving outcomes. The majority of deprescription interventions in primary care are effective. Good communication between healthcare professionals is a key element for success in the deprescription process.

## Background

According to the literature, it has been estimated that polypharmacy affects 30% of individuals aged over 65. Polypharmacy is quantitatively defined as the use of several drugs (usually > 4). In qualitative terms, it refers to the inappropriate use of drugs or use of not clinically indicated drugs [1, 2]. Thus, potentially inappropriate medicines (PIM) are seen to exist amongst different procedures or health technologies. These are estimated to be used by 11.5–62.5% of the elderly population [3, 4]. Polypharmacy and the use of PIM have been associated with more frequent adverse events, lower quality of life and more frequent visits to hospital accident & emergency departments [5–9]. Polypharmacy and PIM expose patients to unnecessary risks and, therefore, efforts to find effective methods for reducing their use should be addressed. A great variety of factors are associated with the discontinuation of treatment such as patient characteristics, patient or care-giver choices, medication-related factors (eg, duration of action, risk–benefit profile, etc.) and practitioner-related or health system-related factors [10].

It is worth noting that the high prevalence of polypharmacy, PIMs and other factors has increased the focus on desprescribing as a separate entity in research and practice [11]. In this regard, and according to the review by Reeve et al. [12], deprescription can be defined as the process of withdrawing an inappropriate medicine, under medical supervision, in order to control polypharmacy and improve outcomes.

The strategies described to examine desprescribing include complete reviews of medication, educational interventions and audits of prescription practices. These interventions have shown some benefits, such as reduced polypharmacy, PIM and costs associated with drug use [13]. Notably, the systematic review conducted by Lucchetti concluded that the most commonly identified PIM prescribed to elderly patients were benzodiazepines, non-steroidal anti-inflammatory drugs (NSAID), antihistamines and antipsychotic drugs [14].

To understand the effectiveness of deprescription interventions, in addition to identifying barriers and enablers to the deprescription process, we performed an umbrella review of the evidence produced by a number of published systematic reviews. In this way, we summarised the evidence of studies assessing non-pharmacological and pharmacological interventions for deprescription in primary care. Further, we described the barriers and enablers of the deprescription process from the patients’ and healthcare professionals’ point of view.

## Methods

An umbrella review of existing systematic reviews was undertaken [15]. The databases consulted were PubMed, Scopus, Embase and The Cochrane Library. This was complemented by searches in the following resources: Google Scholar and the Spanish Network of Agencies for Health Technology Assessment (www.redets.msssi.gob.es/). In addition to this, references in the documents previously identified were reviewed. The search strategy was tailored to the different databases and validated by a librarian who specialised in public health (Additional file 1: Appendix 1). The search was updated in March 2019.

Systematic reviews were included which considered studies that involved programmes or interventions delivered to adults. All reviews had the aim of evaluating deprescribing of one or more regular medication prescriptions by a health professional. Furthermore, systematic reviews that synthesised opinions and beliefs of health professionals or patients about the deprescription process by were also included. We excluded reviews in which > 50% of the included studies were not performed in primary care and were written in languages other than English or Spanish. Included reviews were restricted to primary care, since individuals in institutions, residences or recently discharged from hospital may present widely differing factors (their environment, frailty, nature and number of illnesses and treatment goals). No restrictions were applied based on date of publication. Additional file 1: Appendix 2 contains a more detailed explanation of the stages and methods used in this review.

Data extraction and analysis: two reviewers independently assessed the titles and abstracts to determine whether documents met the inclusion criteria. Those meeting criteria were kept as potential documents (first round of selection). In the second round of selection, three reviewers (AOL, JMM and AMH) independently assessed the full texts of selected documents. In both rounds of selection, any disagreements between reviewers were settled by another reviewer (CBT and AMM) through consensus. A data extraction sheet was designed and, where necessary, authors of reviews were contacted to clarify any doubts which arose. To ensure that the meaning or breadth of data was not changed, particularly in regards to qualitative studies, results were extracted following consensus of the entire team (AOL, AMH, CBT, JMM, AMM and independent staff) (Additional file 1: Appendix 2). The writing process of this umbrella revision was undertaken according to an adapted version of the guide published by Bougioukas [16] (AOL and CBT). Only data on deprescription or data coming from the primary care setting was extracted where possible. Mendeley reference library management software was used for this purpose.

## Results

### Selection of studies and features of reviews

The initial search identified 888 articles. Of these, 734 documents were excluded following a subsequent reading of titles and abstracts. The main reasons for exclusion were that the articles did not report a review study, or that they focused on prevalence, instead of aspects relating to the research question. After reading the full texts of articles, a total of 9 reviews were included in the definitive summary (Table 1), six of which addressed barriers and enablers, whilst the other three explored the effectiveness of interventions (Tables 2 and 3).

**Table 1:** Characteristics of included studies

Review	Objective	Methodology	Results	Conclusion
*Effectiveness of interventions for the deprescription*				
Page (2016) [17]	Determine whether prescription is a safe, effective and feasible intervention for improving mortality and health outcomes amongst the elderly.	Specific database searches of articles published up to February 2015. A qualitative and quantitative summary of the information was made.	A total of 132 articles were included (*n* = 34,143). Observational studies showed that polypharmacy significantly reduced mortality (OR 0.32; 95% CI: 0.17–0.60), but randomized studies did not (OR 0.82; 95% CI 0.61–1.11). Deprescription showed a significant decrease in mortality (OR 0.62; CI 95%: 0.43–0.88) in interventions tailored to patients but not in generalized educational programme (OR 1.21; 95% CI: 0.86–1.69).	Although observational studies suggested that deprescription reduces mortality, this was not observed in randomized studies. When tailored interventions were used, mortality was significantly reduced.
Reeve (2017) [18]	Assess the interventions used to reduce benzodiazepines and Z-drugs, and the impact of these interventions on clinical outcomes in the elderly population.	Database searches for studies carried out with older adults (> 65 years old) between 1995 and 2015. A qualitative and quantitative summary of the information was made.	Seven studies were included. Substitution with melatonin achieved a deprescription rate of 64.3%, with this being 65% in the case of interventions directed by physicians. Interventions which included education and dose reduction (*n* = 2), pharmacological substitution with psychological support (*n* = 1) and dose reduction with psychological support (*n* = 1) showed rates ranging from 27 to 80%.	The deprescription of benzodiazepines is feasible amongst the elderly population, but these rates vary according to the type of intervention. Further studies must be carried out to assess the effectiveness of these interventions.
Hansen (2018) [19]	Evaluate the effectiveness of behaviour change techniques (BCTs) in deprescription interventions	Database searches for studies carried out with older adults (> 65 years old) up to December 2016.	25 studies were included. The number of medications at follow-up was significantly reduced (mean difference − 0.96 95% CI − 1.53, − 0.38; *p* < 0.002). No effects were shown according to intervention type (patient-centred or targeting solely healthcare professionals) or study quality.	BCT deprescription interventions were effective in reducing number of drugs and inappropriate prescribing, but a large heterogeneity in effects was observed.
Dills (2018) [20]	Evaluate the outcome of deprescription compared with standard care.	Database searches for randomized controlled trials involving chronic medical and mental health conditions managed by PCPs	A total of 58 articles were included, two of them were classified as educational interventions (modest deprescription was achieved, without any increase in measured adverse outcomes), twelve were patient drug specific interventions, and six were mixed interventions (educational component plus patient drug-specific interventions, 4 of the 6 studies were successful).	Interventions with the most success in reducing polypharmacy included intense a pharmacist intervention, providing both clinician education as well as patient-specific drug recommendations
Wilsdon (2017) [21]	Determine the effectiveness of interventions for the deprescription of inappropriate proton pump inhibitors amongst the elderly.	Database searches for studies carried out with older adults (> 65 years old) up to January 2017.	21 studies were included. Effective interventions included educating a wide sector of the population and a promotion strategy, providing academic information to GPs, and geriatrician-led prescription for institutionalised patients.	Limited evidence shows that some interventions are more effective than others. There is no evidence that deprescription leads to improved clinical outcomes.
*Barriers and enablers of the deprescription*				
Sirdifield (2013) [22]	Review qualitative studies which explore the experiences and perceptions of physicians about the prescription of benzodiazepines, in order to construct a model explaining the processes underlying current prescription practices.	A search of 7 databases between 1990 and 2011. Results were summarised according to topic.	8 articles were included. Given the limitations of general daily practice prescribing benzodiazepines is complex, awkward and demanding. Physicians face different challenges when initiating, continuing or withdrawing treatment with these drugs.	Results can be used to improve the prescription of benzodiazepines by educating medical professionals about their use and withdrawal, and by improving communication with patients.
Anderson (2014) [23]	Summarise qualitative studies exploring barriers perceived by prescribers, and enablers for minimising the prescription of potentially inappropriate medicines (PIM) to adults.	Searches in specific databases up to March 2014. A qualitative and quantitative summary of the information was made.	21 studies were included (85.7% in primary care). Barriers and enablers for minimising PIM could be grouped into 4 areas: awareness of the problem; secondary inertia, with more inclination to continue rather than cease PIM; self-efficacy as relating to one’s ability to change prescriptions; and the viability of changing prescriptions in patient care environments.	Multiple interdependent factors shape the behaviour of prescribers with regards to continuing or discontinuing a prescription of PIM. Full understanding of barriers and enablers is required in order to develop interventions aimed at reducing PIM consumption and the risk of iatrogenic harm.
Reeve (2013) [24]	Identify barriers and enablers which have an influence on patient decision to stop taking a given medicine.	Database searches for studies carried out with older adults (> 65 years old) up to August 2011.	21 studies were included (1310 participants). 3 areas: agreement with the appropriateness of ceasing medication, lack of a process for ceasing medication, and ‘influences’ on medication cessation. Amongst the barriers and enablers identified was fear of ceasing medication, with the most commonly identified barrier being the appropriateness of ceasing medication.	The decision to cease a given medication is influenced by multifactorial variables. The most common barrier/enabler identified was appropriateness of cessation.
Sirdifield (2017) [25]	Identify and summarise qualitative studies which explore the experiences and perceptions of patients being prescribed benzodiazepines and Z-drugs and, in this way, identify factors which perpetuate the use of these drugs and strategies for achieving safer prescription.	A systematic search in 6 databases for qualitative studies was conducted between January 2000 and April 2014. Results were summarised according to topic.	9 articles were included that addressed 7 topics: (1) negative patient perceptions of insomnia and its impact, (2) failed self-care strategies, (3) initiating search for medical help, (4) attitudes regarding treatment options and service provision, (5) pattern of use variables, (6) withdrawal, (7) reasons for starting or continuing to use.	Psychological dependence, lack of support and denial/unawareness of patients regarding side effects prolongprescription. Educational strategies, increased availability of alternatives and wider, more specific dialogue with patients could support safer prescription.

**Table 2:** Main domains identified in the reviews regarding the practices and perceptions of other health professionals

Anderson (2014) [23]	
**Awareness**: level of insight a prescriber has into the appropriateness of his/her prescribing.	
**Inertia:** failure to act despite awareness that prescribing is potentially inappropriate. This was due to the perception that ceasing PIMs was a less appealing proposition than continuing PIMs.	
**Self-Efficacy**: factors that influence a prescriber’s belief and confidence in his or her ability to address PIM use, involving knowledge, skills, attitudes, influencers, information and support for decision making.	
**Feasibility**: factors that are external to the prescriber and determine the ease or likelihood of change. They relate to patient characteristics, resource availability, work practices, medical and societal health beliefs and culture, and regulations.	
Sirdfield (2013) [22]	
**The changing context of benzodiacepines prescribing**: norms of practice, evidence, guidance, introduction of new drugs and services, legal regulatory frameworks and societal attitudes around the treatment of conditions.	
**Role and responsibility of general practice:** Balance between responsibility over historical prescribing practices (help patient) and the responsibility to minimize benzodiacepine use.	
**The ‘deserving’ patient:** GPs often managed the tension between minimizing prescribing and their responsibility to help patients on a case-by-case basis. They needed to justify giving or withholding benzodiazepines, expressed in the literature through the concept of the ‘deserving patient.	
**Perceived patient expectations** Prescribing was influenced by how doctors perceived patients’ expectations, motivations and ability to cope.	
**GP attitudes towards different interventions** Treatment choices of GPs in response to their perceptions of their patients, their patients’ expectations, and their own role and responsibilities were further influenced by their own attitudes and beliefs about different interventions.	
**Different challenges for managing initiation and withdrawal** GPs’ view of their role, perceived risks and effectiveness of benzodiazepines or alternative treatments, and the patient all influenced whether or not a GP chose to initiate, continue or withdraw benzodiazepines.	
**Ambivalent attitudes towards prescribing benzodiazepines leading to inconsistent strategies for managing prescribing** This attitude ranged from those who rarely prescribed, to those who did not see a problem with prescribing benzodiazepines. For most GPs, located in the middle of this continuum, these were complex decisions leading to conflicting pressures about whether or not to prescribe.

**Table 3:** Main domains identified in included reviews of patient perceptions

Reeve (2013) [24]	
**Appropriateness** Dis/agreement with the appropriateness of cessation. Barrier: Experience and fear of side effects, lack of efficacy, fear of addiction/dependency, etc. Enablers: lack of effectiveness, experience of side effects, fear of addiction.	
**Process**: Barrier: lack of time or support. Enablers: Knowledge that they could restart medication, follow-up/primary care physician support available, physician support (time spent)	
**Influences**: individuals/events that could influence patients’ decisions to cease medication.	
**Fear:** Barrier: fear of cessation (worsening condition, withdrawal reaction, etc.).	
**Dislike:** Enablers: the inconvenience of taking medication, cost of purchasing, etc.	
Sirdifield (2017) [25]	
**Patient’s negative perceptions of insomnia and its impact**: perceptions of insomnia, consequences, etc.	
**Failed self-care strategies:** patients cope with their problems in other ways (distract themselves, lifestyle changes,…)	
**Triggers to medical help-seeking:** medical consultations were triggered by significant life events	
**Attitudes towards treatment options and service provision:** what patients wanted/expected from health professionals.	
**Varying patterns of use:** Although the majority of patients take medicine as prescribed, some of them minimize its use.	
**Withdrawal**: Withdrawal strategies	
**Reason for initial or ongoing use:** the medication was effective in the first instance.	

### Effectiveness of deprescription interventions

Three systematic reviews were included to accomplish the aim of assessing the effectiveness of interventions. The first of them analysed health outcomes amongst patients aged over 65 years old and the nature of the deprescription process for one or more drugs undertaken by a physician [17]. This review included 116 articles but, although 73 of them (62.9%) were carried out in primary care, they did not conduct any specific analyses. Clinical trials on polypharmacy revealed no significant differences in mortality (OR 0.82; 95% CI 0.61–1.11, I^2^ 23%; 10 studies, participants = 3151). Mortality analysis based on intervention type revealed that interventions tailored to patients’ specific situations showed a significant decrease in mortality (OR = 0.62; 95% CI 0.43–0.88, I^2^ 0%; 8 studies, participants = 1906). In contrast, interventions based on education programmes evidenced no change in mortality rates (OR = 1.21; 95% CI 0.86–1.69, I^2^ 0%; 2 studies, participants = 1245). There were no differences based on patient’s age. This was the case for patients older than 80 years (OR = 0.88; 95% CI 0.58–1.34; I^2^ 36%; 7 studies, participants = 1923) and for patients aged between 65 and 79 years (OR = 0.64; 95% CI 0.40–1.04; I^2^ 0%; 3 studies, participants = 1228). In addition, there were no significant differences in mortality in either randomized studies (OR 0.59; 95% CI 0.33–1.07; I^2^ 0%; 5 studies, participants = 453) or non-randomized studies (*p* = 0.81) with single medications/classes. In polypharmacy interventions, deprescription did not cause significant changes in adverse events due to withdrawal, incidence of adverse events, cognitive function, having a fall, or quality of life. Lastly, interventions reduced the number of consumed drugs (MD -0.99; 95% CI − 1.83 to − 0.14; 2 studies, participants = 451), as well as the consumption of potentially inappropriate medicines (MD -0.49; 95% CI − 0.70 to − 0.28; 3 studies, participants = 839). For interventions on a specific drug, no significant differences were observed either in quality of life, or in events caused by medicine withdrawal such as a worsening of the condition with different medicines such as benzodiazepines, glucosamine, carbamazepine, corticoids, etc [17].

Another systematic review evaluated the impact of deprescription interventions and considered the following outcomes: medication load, chronic condition management and mental disorders [20]. Regarding the methods used to assess the deprescription intervention, it was found that interventions with more effective deprescription outcomes were performed by clinicians intensively (showing success in both studies). On the other hand, success was also seen in other studies based on recommending specific medications to patients (success in 4 of 5 studies). Mixed interventions were also successful (educational component with specific medication interventions for high-risk patients) in four of six studies. In this review, some adverse effects of deprescription were identified such as the unmasking of heart failure in diuretics and increases in vertebral fracture in the removal of bisphosphonates.

Further, a review by Hansen et al. [19] evaluated the effectiveness of interventions based on behavioural change techniques. In this way, a meta-analysis including 11 studies found a significant reduction in the average number of prescribed medications − 0.74 (CI 95% -1.26; − 0.22). This result is similar to those found when analysing the 9 studies conducted in primary care − 0,80 (CI 95% -1.40; − 0.21), although these analyses showed significant heterogeneity (*p* < 0.0001).

One systematic review was focused on the deprescription of benzodiazepines (BZD) and Z class hypnotics amongst the elderly (> 65 years old) [18]. This review included a total of 7 studies, 4 of which were carried out in primary care. The first of the primary care studies was a crossover clinical trial in which the intervention involved pharmacological substitution with melatonin and a placebo was administered to 56 patients. The use of melatonin was found to improve quality of sleep (*p* = 0.025), symptoms of depression (*p* = 0.043) and anxiety (*p* = 0.009). Other primary care studies have used mixed methods. In two of these studies patients were given education sessions, informed about harm linked to long-term use of hypnotic drugs and given recommendations on how to reduce BZD use. Further, consumption of benzodiazepines was found to decrease by 27–36%, whilst consumption in the control groups increased by 5 and 4%, respectively. The last of these studies used psychological support to reduce drug doses (*n* = 138), with 80% of patients stopping their treatment after 6 months. Moreover, this study showed an improvement in quality of life (*p* < 0.005) and social skills, although no significant differences were found in sleep quality, cognitive or psychomotor function, mood and symptoms.

Another review focused on deprescription of proton pump inhibitors (PPI) in older adults (≥65 years) and included a total of 21 studies. Interventions were proved to be effective in 6 studies, with 11 being inconclusive and 4 concluding that intervention had been ineffective [21]. With regards to effective interventions, 4 were carried out in primary care with all of these having an educational component, 2 used information leaflets and 2 delivered 30-min educational sessions. In the interventions that used leaflets, a significant reduction was observed both in mean dosage and in the number of patients taking low-dose PPI. The other 2 interventions showed a significant reduction in the risk of continuing to receive potentially inappropriate medicine, both after 12 months (OR 0.40; 95% CI 0.17–0.94) and after 4–6 months (OR 0.30; 95% CI 0.14–0.68; *p* = 0.04). As for the articles reporting inconclusive or ineffective interventions, these referred to two clinical trials carried out in primary care. The first of these determined efficacy of the intervention based on a personalized guide on PPI treatment, prepared by a specialist practitioner and containing instructions for general practitioners (GPs). This study showed that practitioners who received this guide took more action than those who were in the control group, although differences were not significant (*p* = 0.07). The other study assessed the effectiveness of conducting a medication review and preparing an action plan in pharmacists. Although a decrease in the number of inappropriate PPI was observed, differences were not significant (*p* = 0.10). Lastly, it is noteworthy that the remaining articles about primary care included in this review did not conduct any statistical analysis of data.

### Barriers and enablers in the deprescription process according to medical practitioners

We included two reviews in this regard, one on PIM and the other on benzodiazepines and Z class hypnotics. The review on PIM deprescription included 21 articles relevant to older adults (≥65 years), of which 18 were carried out in primary care (85.7%) [23]. Poor perceptions held by prescribing physicians of prescription appropriateness was shown to be a barrier, concluding that it is necessary for prescriptions at a population level to be translated into prescription practices at an individual level. Additionally, another considered barrier was the perception that it may be of more value to continue PIM treatment rather than suspend it, even though such treatment could be inappropriate. Barriers were identified relating to the consequences of deprescription for prescribers (increased burden of care, conflicts with colleagues, reduced credibility, etc.), patients (withdrawal symptoms, worsening of the event/condition that triggered treatment in the first place, etc.) and other professionals (increased workload, etc.). In contrast, taking the benefits of deprescription into consideration is an enabler. Thus, underestimating the adverse effects of medicines was identified as a barrier, particularly in the case of psychoactive drugs. Another identified barrier was delegating the responsibility for deprescription to another professional.

In this regard, having better information about the risk-benefit ratio, greater confidence to cease treatment, and more experience and training were identified as enablers for deprescription. Gaps in knowledge and skills led to poor communication between the different healthcare professionals involved in patient care (inadequate transfer, fragmented information, etc.). This barrier is closely linked to complaints voiced by healthcare professionals in relation to lack of time and not being able to access all the information required. In this respect, pressure to comply with the recommendations of clinical practice guidelines and the routine effect of prescription are negatively perceived by professionals. Moreover, with regards to the feasibility of change, the main barriers mentioned were resistance or ambivalence of patients to change and their refusal to try alternatives. In terms of resources, the most commonly found limitation was lack of time and effort to review/cease medication, followed by the limited availability of alternatives. Another perceived enabler was access to support services (mental health workers and pharmacists) in order to review medical prescriptions. Feelings of discomfort and reluctance to question the prescription decisions made by a colleague (resulting in repeated prescription) were linked to medical and social beliefs. Such responses came out of respect for professional independence or the medical hierarchy. Lastly, externally imposed quality guidelines such as prescription thresholds (for example, restricted access, cost, etc.) and monitoring by the prescription authorities are also perceived as barriers. Greater dialogue with patients increases patient’s understanding and facilitates shared decision making [23].

The review of benzodiazepines and Z class hypnotics included 8 studies which analysed general practice and nurse experiences [22]. Although primary care practitioners valued being better informed about risks, the historical culture of optimism regarding benefits and ingenuity regarding risks, has been replaced by a more sceptical attitude which could be a barrier for deprescription. Physicians mentioned treating more patients, including those with mental morbidity, who used to be treated in specialised care. Whilst GPs generally feel responsible for these inappropriate prescriptions, different attitudes are observed regarding historical prescriptions. It must be pointed out that the conditions for prescribing benzodiazepines and for other interventions, including deprescription, are dictated by patient characteristics such as their age, drug abuse, complexity, etc... However, these circumstances are conditioned by the practitioner’s knowledge or empathy with the patient’s situation. Other mentioned factors include lack of time, possible patient dissatisfaction and concern that patients may change their GP, all of which complicates deprescription of these medicines.

With regards to the types of strategies used for managing the prescription of benzodiazepines, a wide variety of situations can be considered. Three main situations are described: practitioners prefer short-term, low-dose prescriptions and warn their patient about potential adverse effects; practitioners consider patient demedication as part of their remit; and practitioners who tacitly and explicitly rule out benzodiazepine use (Table 2).

### Barriers and enablers in the deprescription process according to patients

A review by Reeve et al. included 21 studies (1310 participants) [24] and described the barriers and enablers that may influence patient’s decisions to cease medication. The most frequently mentioned aspect in relation to barriers is appropriateness. In this sense, patients question the withdrawal of their medication, arguing that they experienced improvement when treatment began and, even if this improvement does not continue, they hope that it will benefit them in the future. Moreover, failure when using other alternatives was described in this study. Furthermore, lack of support, and lack of time and opportunity for discussion with the primary care physician was mentioned as a difficulty before and after deprescription. The fact that patients and physicians have a good relationship results in a positive influence of GPs on the cessation of treatment, whilst also encouraging GP support for increasing patient’s disposition towards leaving treatment. Similarly, the fact that their medical practitioner continues to prescribe is perceived as an indication that continued treatment is necessary. Most of the included studies refer to non-specific fears such as “worrying about the negative consequences of treatment withdrawal”, “seeing treatment withdrawal as problematic” and “being unsure of one’s ability to cope with it”. Other expressed fears were concerns that the disease may worsen or experiences of dependency syndromes.

Deprescription enablers were found in the case of patients who felt that they no longer needed to take their medication, questioned it, found it ineffective or were concerned about its adverse effects. Another deprescription enabler (or enabler of reduced dosage) was knowing that, if necessary (e.g. symptoms reappear), individuals could start taking the medication again and knew that they could count on the support of medical and other services. A good patient-physician relationship, the media and publication of new evidence were identified as positive influences. The inconvenience of taking medicines (number of tablets or injections each day), in addition to the cost, were mentioned as reasons to stop taking them. In addition, patients expressed psychological benefits of stopping to take medication, reporting having more control over their lives and liking themselves better when not on medication [25] (Table 3).

## Discussion

To the extent of our knowledge, this umbrella review was the first attempt to provide a general overview of the deprescription process in primary care, taking into consideration both the efficacy of interventions, alongside barriers and enablers of the deprescription process. The articles reviewed showed that deprescription in clinical practice was feasible and reduces the quantity of both medicines and PIM use [26]. Additionally, knowledge of relevant barriers and enablers allows better implementation of interventions for deprescribing medicines. Most of the included reviews showed that communication and support amongst healthcare professionals are proved to be key elements for optimising the deprescription process.

In this respect, we have to take into account that the benzodiazepine group, both anxiolytic (A) and hypnotic (H) drugs alike, is one of the most prescribed drugs in the majority of developed countries, with Europe having the highest mean consumption worldwide [27, 28]. Furthermore, scientific literature states that consumption of benzodiazepines is linked to an increased risk of accidents, suicide, mortality, dementia, and broken hips [29–32]. This justifies why 3 of the 7 reviews included in this study focus on these drugs.

The patient-centred review of benzodiazepines shows that the adverse effects of medication can have an effect on the use of these drugs. However, some patients state that they are willing to pay this price if it means they will experience improved sleep quality. The main reasons for deprescription are being aware of how medication affects patients’ daily routines and those of the people around them [25].

The deprescription process encourages reflection on a number of issues [33]. The first is about the appropriateness of treatment (the principle of beneficence), in view of the negative or neutral risk-benefit ratio of PIM. The second regards patient safety (the principle of non-maleficence), in view of the fact that many patients taking these treatments are exposed to unjustified risks and adverse effects. The third issue refers to efficiency (the principle of fairness) and speaks to making a better use of scarce resources [34]. Furthermore, it must be borne in mind that prescription decisions are not made in isolation. Indeed, such decisions are taken in careful consideration of a large number of individual factors such as general state of health, care objectives and compliance with the current system [35]. In this respect, the literature uncovers one factor that promotes less-than-optimal use of health technologies. This factor is inadequate communication between physicians and patients. This lack of communication is linked to false expectations about benefits and choosing treatments which might not have been chosen had better information been available [36].

From the perspective of patient safety, the results of the present work are of particular relevance. In accordance with an examination of adverse effects in primary care, one systematic review estimated the prevalence of medication errors in community environments to fall between 2 and 94%. This disparity may be due to differences in definitions, populations, methodologies, etc. [37]. In Spain, it has been estimated that 48.2% of the adverse effects identified are related to medication [38, 39]. This phenomenon is particularly relevant in polypharmacy amongst elderly patients [40–42]. In this respect, a systematic review calculated the cost of patient non-safety and adverse effects in the administration of medicines. This determined costs of between 469 and 790 million euros annually for medication-related adverse effects, and just over 91 million euros annually for incorrect medication [43].

An outstanding finding of this review is that interventions tailored to patients’ specific situations produce the best results for deprescription as they decrease therapeutic load [20]. This fact underlines the importance of focusing interventions to target patient characteristics. Moreover, these interventions should have multiple components (advice, communication, ongoing support, etc.). In this regard, neither protecting patient independence, nor taking their preferences into account, should be discarded. Over recent decades, various tools have been developed that facilitate the identification of inappropriate prescription in elderly patients. Examples include deprescribe.org, which has several tools (including an app), and provides information for professionals and patients [44]. Another useful tool is the STOPP criteria (Screening Tool of Older Person’s potentially inappropriate Prescriptions). This consists of 65 indicators of potentially inappropriate prescriptions which include drug-medication and medication-clinical interactions, therapeutic duplicity and medications that increase the risk of cognitive impairment and falls in the elderly [45]. Finally, the AGS Beers Criteria is another tool for identification of inappropriate prescription, although its implementation in Europe is limited by its lack of applicability and some other deficiencies [46, 47].

### Limitations

This review has some potential limitations which must be taken into account. There may be a risk of misrepresenting incomplete evidence, however, to minimise this, different databases were consulted. Furthermore, a systematic search strategy was used to make it more likely that the included reviews were representative of the body of systematic reviews available on deprescription interventions effectiveness. Umbrella reviews can only report what researchers have investigated, published and systematically reviewed or meta-analysed [48]. Finally, although the information extracted was focused on primary care, some of the included systematic reviews were applied to other settings in addition to primary care. Consequently, results pertain to an array of contexts and patients and, therefore, may not be applicable to some specific patient populations (e.g. frail older people).

## Conclusions

In general, most of the deprescription interventions carried out in primary care are effective. In addition, the gathered evidence showed that, in certain cases, deprescription has benefits for patients. In terms of barriers and facilitators, good communication between healthcare professionals was as a key element for success in the deprescription processes, with these elements being similar to those described in other areas [49]. In the same way, interventions designed to promote the rational use of medicines should provide information about the risk-benefit ratio, considering the possibility that the resultant risks exceed the potential benefits [50, 51].

## Supplementary information

**Additional file 1: Appendix 1.** Complete search strategy by database. **Appendix 2:** Investigation stages and methods for umbrella review.

## Data Availability

Not applicable.

## Abbreviations

BZD: Benzodiazepines
95% CI: 95% confidence intervals
NSAID: Non-steroidal anti-inflammatory drugs
OR: Odds ratio
PIM: Potentially inappropriate medicines
