# Supplementary material for: Evidence for deprescription in primary care through an umbrella review
Source: BMC Fam Pract. 2020 Jun 8;21:100. doi: 10.1186/s12875-020-01166-1 (PMC7278115; doi:10.1186/s12875-020-01166-1)
Supplement: Supplementary file 1 — Additional file 1: Appendix 1. Complete search strategy by database. Appendix 2: Investigation stages and methods for umbrella review. [file 12875_2020_1166_MOESM1_ESM.docx]

## Appendices

### Appendix 1. Complete search strategy by database

**Strategy 1 (PubMed)**

#5,"Search #1 AND #4

#4,"Search #2 OR #3

#3,"Search ((prescri*[title/abstract] OR medication*[title/abstract] OR drug*[title/abstract] OR medicine*[title/abstract]))) AND (inappropriate*[title]) OR Deprescri*)

#2,"Search "Inappropriate Prescribing"[Mesh] OR "Inappropriate Prescribing" OR "Deprescriptions"[Mesh] OR "deprescriptions"

#1,"Search (("Primary Health Care"[Mesh] OR "Primary care" OR “general practice”[MeSH] OR “general practitioners”[MeSH] OR "Family Practice"[MeSH] OR "Physicians, Family"[Mesh] OR "family doctor" OR "family physician" OR "family practitioner"

**Strategy 2 (Ovid)**

1 prescri$.ab,ti.

2 medication$.ab,ti.

3 drug$.ab,ti.

4 medicine$.ab,ti.

5 pharmaceutical preparations.ab,ti.

6 inappropriate$.ab,ti.

7 Deprescri$.ab,ti.

8 1 or 2 or 3 or 4 or 5

9 6 or 7

10 8 and 9

11 Inappropriate Prescribing/

12 Inappropriate Prescribing.ab,ti.

13 Deprescriptions/

14 deprescription.ab,ti.

15 11 or 12 or 13 or 14

16 10 or 15

17 "Primary care".ab,ti.

18 Family Practice/

19 family doctor.ab,ti.

20 family physician.ab,ti.

21 family practitioner.ab,ti.

22 exp Primary Health Care/

23 physicians, family/ or physicians, primary care/

24 exp General Practice/

25 general practitioners/

26 17 or 18 or 19 or 20 or 21 or 22 or 23 or 24 or 25

27 16 and 26

28 exp Anti-Infective Agents/

29 Anti Infective Agents.ab,ti.

30 antimicrobial agents.ab,ti.

31 28 or 29 or 30

32 27 not 31

33 Meta-Analysis as Topic/

34 meta analy$.tw.

35 metaanaly$.tw.

36 Meta-Analysis/

37 (systematic adj (review$1 or overview$1)).tw.

38 exp Review Literature as Topic/

39 or/33-38

40 cochrane.ab.

41 embase.ab.

42 (psychlit or psyclit).ab.

43 (psychinfo or psycinfo).ab.

44 (cinahl or cinhal).ab.

45 science citation index.ab.

46 bids.ab.

47 cancerlit.ab.

48 or/40-47

49 reference list$.ab.

50 bibliograph$.ab.

51 hand-search$.ab.

52 relevant journals.ab.

53 manual search$.ab.

54 or/49-53

55 selection criteria.ab.

56 data extraction.ab.

57 55 or 56

58 Review/

59 57 and 58

60 Comment/

61 Letter/

62 Editorial/

63 animal/

64 human/

65 63 not (63 and 64)

66 or/60-62, 65

67 39 or 48 or 54 or 59

68 67 not 66

69 32 and 68

**Appendix 2:** Investigation stages and methods for umbrella review.

| **Step** | **Purpose** | **Activities** | **Persons responsible** |
| --- | --- | --- | --- |
| 1 | Determining the question | Several meetings were held in order to agree on the definitions and objectives of this review. Based on the literature, key search terms and possible sources of literature for searching were identified. First of all, and taking into account search results and the objectives of this study, we decided that the ideal method for summarising evidence was to make an umbrella review. | AOL, CBT, AMH, JMM, AMM and independent staff ^*^ |
| 2 | Identification of relevant studies | The preliminary criteria for including and excluding studies were developed and reviewed iteratively. To maximise search sensitivity, two searches were carried out in parallel, with and without review filter (SIGN). The literature search was complemented by other databases and grey literature, and was finished on 27 September 2017 and updated on march 2019 using Mendeley documentation management software. | AOL and JMM |
| 3 | Selection of studies | Titles and abstracts were reviewed independently, taking into account the pre-determined inclusion and exclusion criteria. Then the full text of articles which were relevant for meeting objectives was reviewed, and any disagreements solved by consensus. | AOL, AMH, CBT, JMM and AMM |
| 4 | Extraction of information and drafting results | Information extraction was done by the entire team on a consensus basis. Then an independent person checked that the information extracted was complete and precise. Information in the studies identified was then categorised, and subsequently the paper was drafted. | AOL, AMH, CBT, JMM, AMM and independent staff |
| 5 | Participation of interested parties | The results of this review form part of a larger project being carried out on “do not” recommendations based on scientific evidence. Also being carried out is an in-depth analysis using mixed methods. It must be pointed out that presentations will be given both to different healthcare professionals and to policy makers. | The entire team |

*The search strategy was checked by a librarian with ample experience in the public health sphere.
